# Palmitoylethanolamide: A Nutritional Approach to Keep Neuroinflammation within Physiological Boundaries—A Systematic Review

**DOI:** 10.3390/ijms21249526

**Published:** 2020-12-15

**Authors:** Stefania Petrosino, Aniello Schiano Moriello

**Affiliations:** 1 Endocannabinoid Research Group, Istituto di Chimica Biomolecolare, Consiglio Nazionale delle Ricerche, Via Campi Flegrei 34, 80078 Napoli, Italy; aniello.schianomoriello@icb.cnr.it; 2 Epitech Group SpA, Via Einaudi 13, 35030 Padova, Italy

**Keywords:** dietary food, endocannabinoid system, mast cells, neuroinflammation, nutrient, palmitoylethanolamide

## Abstract

Neuroinflammation is a physiological response aimed at maintaining the homodynamic balance and providing the body with the fundamental resource of adaptation to endogenous and exogenous stimuli. Although the response is initiated with protective purposes, the effect may be detrimental when not regulated. The physiological control of neuroinflammation is mainly achieved via regulatory mechanisms performed by particular cells of the immune system intimately associated with or within the nervous system and named “non-neuronal cells.” In particular, mast cells (within the central nervous system and in the periphery) and microglia (at spinal and supraspinal level) are involved in this control, through a close functional relationship between them and neurons (either centrally, spinal, or peripherally located). Accordingly, neuroinflammation becomes a worsening factor in many disorders whenever the non-neuronal cell supervision is inadequate. It has been shown that the regulation of non-neuronal cells—and therefore the control of neuroinflammation—depends on the local “*on demand*” synthesis of the endogenous lipid amide Palmitoylethanolamide and related endocannabinoids. When the balance between synthesis and degradation of this bioactive lipid mediator is disrupted in favor of reduced synthesis and/or increased degradation, the behavior of non-neuronal cells may not be appropriately regulated and neuroinflammation exceeds the physiological boundaries. In these conditions, it has been demonstrated that the increase of endogenous Palmitoylethanolamide—either by decreasing its degradation or exogenous administration—is able to keep neuroinflammation within its physiological limits. In this review the large number of studies on the benefits derived from oral administration of micronized and highly bioavailable forms of Palmitoylethanolamide is discussed, with special reference to neuroinflammatory disorders.

## 1. Introduction

Modern neurosciences consider neuroinflammation as “any inflammatory process, both acute and chronic, that affects the central or peripheral nervous system” [1]. Neuroinflammation is a widely studied issue since it may be induced by—and associated with—certain pathological clinical conditions that may result in neurodegeneration [2]. Immune cells [3,4], like mast cells and microglia [5,6], as well as astrocytes (i.e., neuroglial cells that do not belong to the immune system [7]) are importantly involved in neuroinflammatory processes. These cells are collectively known as “non-neuronal cells” in relation to their lineage and location, since they are not neurons albeit they are associated with—or located within—the peripheral and central nervous system. Under physiological conditions, non-neuronal cells support the well-being and well-function of neurons through diverse functions, including neurotrophic factor secretion, and they are thus able to ensure the homodynamic balance of the nervous system, i.e., the dynamic homeostatic process aimed at coping with different challenges [8]. On the contrary, once hyper-activated they can profoundly affect neuronal responses, in particular pain signaling systems and neurodegenerative pathways [9,10]. Actually, it has emerged that hyper-activated non-neuronal cells mutually interact with each other through a cytokine-mediated cross-talk that can amplify or chronicize neuronal suffering [11,12]. If uncontrolled, non-neuronal cells are thus considered to play an important role in the induction and maintenance of peripheral and central sensitization, associated with inflammatory pain, chronic and neuropathic pain, and with many clinical conditions of dysmetabolic, traumatic, or degenerative nature [1,13,14]. Non-neuronal cells are actually recognized a crucial trait d’union between systemic inflammation and the brain [15,16,17]. Systemic increase of inflammatory mediators (e.g., cytokines) significantly contributes to neuroinflammatory reactions [18,19]. Damage to the blood brain barrier and increased entrance of proinflammatory mediators and immune cells into the brain are the main consequences and result in the activation of non-neuronal cell and hence neuroinflammation [17,18]. COVID-19 associated neurological disease (i.e., encephalitis) without evidence of central nervous system viral invasion is a current example of how systemic hyperinflammation (i.e., virus-induced strong systemic cytokine storm) can activate neuroinflammatory cascade at the central nervous system level [20,21].

On the basis of the aforementioned scientific knowledge, several research groups have focused their attention on the down-regulation of non-neuronal cells as a potential mean to control neuroinflammatory and neurodegenerative disorders, as extensively reviewed recently [22,23,24]. To this aim, the hypothesis of using Palmitoylethanolamide (PEA) has been put forward, given its ability to physiologically regulate non-neuronal cells [25,26]. In mammalian species, PEA is produced “*on demand*” by different cell types [27,28,29] in response to actual or potential damage for protective purposes [30]. An ever-growing body of evidence indicates that PEA plays a key role in restoring homodynamic balance during disease conditions [31]. In particular, PEA is a promising lipid signaling molecule involved in the physiological program of resolution, i.e., the coordinated and dynamic control of inflammation aimed at averting the occurrence of non-resolving inflammation [32]. PEA has been detected in several tissues and body fluids, e.g., the brain and spinal cord [33,34,35,36,37,38,39,40], eye and cardiac tissues [41,42,43,44], liver and testicles [45,46,47], gastrointestinal system [48,49], skin, muscle [50,51,52,53] and blood [54,55,56,57]. In addition, PEA is normally present in the amniotic fluid, umbilical vein, and artery (i.e., the main sources of fetal nutrition), as well as human breast milk [47,58], and is thus considered a genuine early nutrient. Importantly, PEA was shown to be protective toward perinatal asphyxia [59] and critical for the healthy development of the fetal nervous system [60]. Furthermore, PEA occurs in several food sources, albeit at low levels (Table 1). In particular, it was originally detected in egg yolk [61,62], and subsequently in peas, tomato, beans, peanuts, soya lecithin [63,64], and in cow’s milk [65]. Recently, PEA has also been identified in coffee and their respective infusions [66]. Accordingly, it can be speculated that under physiological conditions appropriate levels of PEA can be obtained through breastfeeding in the newborns and a balanced diet in the adulthood, besides a suitable “*on demand*” body production. On the contrary, in diseased conditions associated with neuroinflammation, one might envision that PEA endogenous production is insufficient to fully exert its protective role [67]. This is the case of pathological settings characterized by microglial activation, like neuropathic pain, where spinal and/or supraspinal levels of PEA are severely decreased [39,68]. Conceivably, raising PEA levels may be a promising therapeutic strategy in the control of neuroinflammation [32]. In line with this view, inhibition of PEA catabolic enzyme in experimental systemic inflammation results in a significant elevation in the endogenous PEA levels in the brain and an associated decrease of brain inflammatory mediators [69]. Likewise, increasing the levels of PEA in LPS-challenged microglial cells through the inhibition of its hydrolysis significantly reduces microglial activation [70]. On the other hand, administration of PEA significantly relieves neuroinflammatory-associated disorders [32], and counteracts neuroinflammation at the cellular level, provided the compound is formulated in bioavailable forms [71].

The highly lipophilic nature of PEA, in fact, limits its dissolution and absorption as well as the bioavailability for achieving health benefits by oral route. Bioavailable formulations of PEA, i.e., micronized PEA (PEA-m) and ultra-micronized PEA (PEA-um) with a particle size distribution in the 2–10 μm and 0.8–6 μm range, respectively, have been developed accordingly [71,75]. After oral administration, PEA-m and PEA-um have repeatedly shown higher bioavailability and superior effects compared to naïve PEA [75,76]. PEA-um also penetrates into the central nervous system, as shown by the significant increase of PEA levels in the hippocampus, brain, and spinal cord after oral administration of PEA-um [76,77]. Finally, PEA-m and PEA-um have shown favorable safety profiles with no evidence of toxicity [78]. PEA-m and PEA-um are used in the formulation of products that are currently classified as Foods for Special Medical Purposes (FSMP) under the EU Regulation. The products are intended to meet part of the nutritional requirements of patients with specific diseases and clinical conditions sustained by neuroinflammation. In this review, we focus on the particular relevance of uncontrolled neuroinflammation in the onset of central and peripheral neurodegenerative disorders. The large number of studies on the beneficial effects of PEA-m and PEA-um in pre-clinical disease models as well as naturally occurring disorders are discussed from this perspective (Table 2 and Table 3). Moreover, given the role played by oxidative stress in neuroinflammation [79], we also focus on the advantage of combining PEA-um with polyphenolic antioxidant substances of vegetable origin [80], such as luteolin [81,82] and polydatin [83] (Table 2 and Table 3). However, inasmuch as it would be unmanageable to describe here all pathological conditions in which PEA-m and PEA-um (alone or in combination with luteolin or polydatin) have been administered, we place our attention only on some neurodegenerative and neurological disorders, as well as pain syndromes sustained by neuroinflammation.

## 2. Endogenous PEA: Metabolic Pathways and Mechanisms of Action

**Figure 1:**
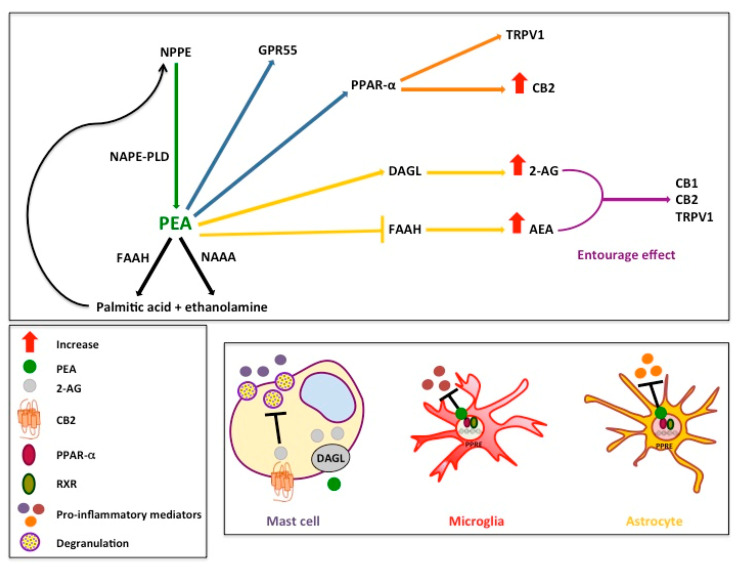
Metabolic pathways and mechanisms of action of PEA. PEA is synthetized by NAPE-PLD (green arrow) and hydrolyzed to palmitic acid and ethanolamine by FAAH and NAAA (black arrows) [28]. PEA directly activates GPR55 [149] and PPAR-α receptors (blue arrows) [148]. PEA activates TRPV1 receptors [145,146] and increases the expression of CB2 receptors (orange arrows) via direct activation of PPAR-α receptors (blue arrow) [154]. PEA, through the stimulation of the activity of DAGL [143] or the inhibition of the expression of FAAH (yellow arrows) [142], increases the endogenous levels of 2-AG and AEA, respectively, which directly activate CB1, CB2, and TRPV1 receptors (“entourage effect”) (violet arrow) [57,142,143,145,146]. PEA, possibly through an allosteric modulation of TRPV1 receptors, potentiates the actions of AEA and 2-AG at TRPV1 receptors (“entourage effect”) [57,145,146]. PEA inhibits the activation of mast cells through an indirect CB2-mediated mechanism (i.e., increased 2-AG synthesis) [140,143]. PEA reduces the activation of microglia and astrocytes through a PPAR-α-mediated mechanism [155,156]. Abbreviations: 2-AG, 2-arachidonoylglycerol; AEA, anandamide; CB1, type-1 cannabinoid receptors; CB2, type-2 cannabinoid receptors; DAGL, diacylglycerol lipase; FAAH, fatty acid amide hydrolase; GPR55, G-protein coupled receptor 55; NAAA, *N*-acylethanolamide hydrolyzing acid amidase; NAPE-PLD, *N*-acyl-phosphatidyl-ethanolamine-selective phospholipase D; PEA, palmitoylethanolamide; PPAR-α, peroxisome proliferator-activated receptor-α; TRPV1, transient receptor potential vanilloid type-1.

The body production of PEA mainly depends on a membrane phospholipid precursor, namely C:16 *N*-acyl-phosphatidyl-ethanolamine (NAPE) or *N*-palmitoyl-phosphatidyl-ethanolamine, which is then hydrolyzed to generate PEA by the action of NAPE-selective phospholipase D (NAPE-PLD) [134]. The degradation of PEA is instead catalyzed by the action of fatty acid amide hydrolase (FAAH) [135] and *N*-acylethanolamide hydrolyzing acid amidase (NAAA) [136], leading to the formation of palmitic acid and ethanolamine, which are fully reused by cells to synthesize the membrane phospholipids [137,138]. The mechanism through which PEA maintains the normal reactivity of mast cells and microglia/astrocytes in the peripheral and central nervous system respectively is historically known as autacoid local injury antagonism (ALIA) [139,140,141]. It is now acknowledged that ALIA depends on the ability of PEA to (i) indirectly interact with the type-1 (CB1) and type-2 (CB2) cannabinoid receptors [142,143], (ii) exert a positive allosteric modulation of the transient receptor potential vanilloid type-1 (TRPV1) [57,144,145,146,147], (iii) directly interact with the peroxisome proliferator-activated receptor-α (PPAR-α) [148] or with the orphan G-protein coupled receptor 55 (GPR55) [149,150]. The indirect interaction of PEA with specific receptors of the endocannabinoid and endovanilloid systems is well-known as the “entourage effect” [142,151]. By inhibiting the expression of the enzyme hydrolyzing the endocannabinoid anandamide (AEA) [142], or by stimulating the activity of the enzyme biosynthesizing the endocannabinoid 2-arachidonoylglycerol (2-AG) [143], PEA increases the endogenous levels of these lipid mediators and potentiates their actions at CB1, CB2, and TRPV1 receptors [57,142,143,145,146]. Nevertheless, it has also been demonstrated that PEA via direct interaction with PPAR-α receptors is capable to activate TRPV1 receptors [152,153], as well as to increase the expression of CB2 receptors [154]. These discoveries revealed interesting multiple and synergistic mechanisms of action of PEA, making it able to exert multiple effects and act on different cell types in both the central and the peripheral nervous system (Figure 1).

## 3. Pre-Clinical and Clinical Effects of PEA in Micronized and Co-Micronized Formulations on Neuroinflammation Associated with Neurodegenerative and Neurological Disorders

### 3.1. Parkinson’s Disease 

Under physiological conditions, non-neuronal cells like microglia and astrocytes support the well-being and well-function of the brain through diverse functions, including neurotrophic factor secretion. On the contrary, in the course of Parkinson’s disease (PD) a shift from neuroprotective to neuroinflammatory phenotype occurs and the non-neuronal cells sustain disease onset and progression [157,158,159]. The efficacy of PEA in controlling neuroinflammation-associated neurodegeneration has been demonstrated in an animal model of PD induced by 1-methyl-4-phenyl-1 2 3 6-tetrahydropyridine (MPTP). Chronic treatment with PEA (10 mg/kg for 7 days) protected against MPTP-induced loss of tyrosine hydroxylase neurons, reduced MPTP-induced microglial activation and the number of glial fibrillary acidic protein (GFAP)-positive astrocytes [84]. Moreover, PEA exerted a positive effect on the cognitive and motor deficits, which was shown to be mediated at least in part by PPAR-α receptor [84]. A formulation containing PEA co-ultra-micronized with luteolin (co-ultraPEA-Lut) has been investigated in the same animal model of PD. The administration of co-ultraPEA-Lut (for 7 days at 1 mg/kg dose) re-established the normal expression of the enzyme tyrosine hydroxylase, and most importantly, reduced the increased expression of GFAP (a marker of astrocyte activation), inducible nitric oxide synthase (iNOS), and pro-inflammatory cytokines in the brain [85]. In a further study performed on MPTP-induced PD in aged mice, chronic pre-treatment with PEA-m (10 mg/kg for 60 days) was found to counteract the behavioral deficits, the reduced expression of tyrosine hydroxylase and dopamine transporter, as well as the up-regulation of α-synuclein and β3-tubulin in the substantia nigra [80]. Importantly, PEA-m also reduced the expression of pro-inflammatory cytokines and showed a pro-neurogenic effect in the hippocampus [80]. A different animal model of PD was also used to investigate the protective effect of PEA against PD-associated neuroinflammation. In particular, the disease was induced by 6-hydroxydopamine (6-OHDA) and PEA was administered at 3, 10, or 30 mg/kg for 28 days [86]. Results showed that PEA was able to (i) improve 6-OHDA-induced behavioral impairments, (ii) increase tyrosine hydroxylase expression, (iii) exert an anti-apoptotic effect and, most importantly, (iv) reduce oxidative stress and neuroinflammation (i.e., reduced iNOS and cyclooxygenase-2 (COX-2) expression) [86]. The observed effects were PPAR-α receptor-mediated [86]. These pre-clinical studies suggest that PEA-m and PEA-um (alone or in association with Luteolin) are effective in improving PD motor function through mechanisms aimed at controlling neuroinflammation and protecting neurons. Accordingly, the clinical utility of FSMP containing PEA-m has been evaluated in a study conducted on 30 patients affected by PD [87]. Add-on oral administration of PEA-um (3 months at 600 mg/bid followed by 600 mg/die for 12 months) to PD patients receiving levodopa therapy produced a significant and progressive reduction in the total Movement Disorder Society/Unified Parkinson’s Disease Rating Scale (MDS-UPDRS) score (parts I, II, III, and IV) [87]. In other words, oral supplementation with PEA-um slowed down the disease progression and disability scores, and proved to be a valuable add-on option in PD patients [87].

### 3.2. Alzheimer’s Disease

The efficacy of PEA in controlling neuroinflammation and the associated neurodegeneration has also been demonstrated in several pre-clinical models of Alzheimer’s disease (AD). In particular, in an animal model of AD, consisting of intracerebroventricular injection of beta amyloid (Aβ) 25–35 peptide, chronic treatment with PEA (10 or 30 mg/kg once a day for 1 or 2 weeks), starting 3 h after Aβ 25–35 peptide injection, reduced (10 mg/kg) or prevented (30 mg/kg) learning and memory deficits, lipid peroxidation, iNOS induction, and caspase3 activation induced by Aβ 25–35 peptide [88]. Again, the effects were PPAR-α receptor-mediated [88]. In a similar animal model of AD, consisting of intrahippocampal injection of Aβ 1–42 peptide, PEA (10 mg/kg once day for 7 days) was likewise shown to counteract the memory-impairing effect, amyloidogenesis, tau protein hyperphosphorylation, and reactive gliosis induced by Aβ 1–42 peptide, through the involvement of PPAR-α receptors [89]. In a different animal model of AD, namely in young (6-month-old) and adult (12-month-old) triple transgenic AD (3×Tg-AD) mice, the anti-neuroinflammatory effects of PEA-um have also been demonstrated [90]. Chronic treatment with PEA-um (10 mg/kg) for 3 months was able to normalize astrocyte function [90,91]. Improvement of learning and memory, decreased depressive and anhedonia-like behaviors, as well as reduced Aβ formation and tau protein phosphorylation were also observed [90,91]. Finally, PEA-um promoted neuronal survival in the CA1 sub-region of the hippocampus [90,91]. The observed effects (e.g., reduced reactive astrogliosis and restored neuronal trophic support) were superior in younger mice as compared to older mice, suggesting that PEA-um may be a promising strategy to slow AD progression in the early stages of the disease [90,91]. More recently, in the same 3×Tg-AD model, chronic oral administration of PEA-um (100 mg/kg/day for 3 months) also rescued cognitive deficit and decreased the hippocampal level of extracellular glutamate [77]. Again, a significant effect on neuroinflammation was observed, as shown by the almost complete inhibition of interleukin (IL)-6 increase in the hippocampus, and the reduced the production of ROS [77]. Evaluation of co-ultraPEA-Lut has also been reported in the early stage of AD. In particular, the animals received co-ultraPEA-Lut (5 mg/kg/day) for 2 weeks starting on the day of intrahippocampal Aβ 1–42 peptide injection [92]. Chronic administration of co-ultraPEA-Lut prevented the Aβ-induced astrogliosis and microgliosis and the upregulation of iNOS, COX-2, IL-1β, tumor necrosis factor (TNF)-α, and IL-6 gene expressions [92]. Moreover, the treatment normalized the downregulated IL-10 mRNA levels, demonstrating clear anti-neuroinflammatory properties [92]. Although clinical studies on PEA formulations in AD patients are still missing, a case report of a 67-year-old woman affected by mild cognitive impairment (MCI) and administered co-ultraPEA-Lut (700 mg + 70 mg once daily) has recently been described [93]. MCI is the stage between the expected cognitive decline of normal aging and the more serious decline of dementia, hence recognized as a risk factor for AD. At baseline, the patient presented a mild memory impairment—as demonstrated by specific neuropsychological assessments—as well as a bilateral hypo-perfusion in different brain areas at single-photon emission computed tomography (SPECT) [93]. After 3-months supplementation a mild (although non-significant) cognitive amelioration was recorded, whereas 3 months later the neuropsychological evaluation was almost normal with a significant improvement of Rey Auditory Verbal Learning Test (RAVLT), Attentive Matrices (AM), and Trail Making Test (TMT) compared to basal conditions [93]. Moreover, the brain SPECT was almost within the normal range [93]. These promising results suggest that dietary supplementation with co-ultraPEA-Lut might be a valuable option in the management of MCI-associated neuroinflammation and related neurodegenerative disorders.

### 3.3. Multiple Sclerosis 

Uncontrolled neuroinflammation is a widely recognized hallmark of Multiple sclerosis (MS) [160,161]. In an animal model of MS, i.e., the Theiler’s Murine Encephalomyelitis Virus-Induced Demyelinating Disease (TMEV-IDD), the administration of PEA (5 mg/kg daily for 10 days, started 60 days post-infection) counteracted the motor deficits associated with the disease, and exerted an anti-neuroinflammatory effect by reducing the expression of pro-inflammatory cytokines and decreasing microglial activation [94]. In a different animal model of MS, the Experimental Autoimmune Encephalomyelitis (EAE) based on active immunization with a fragment of Myelin Oligodendrocyte Glycoprotein (MOG_35–55_), the administration of co-ultraPEA-Lut (5 mg/kg from 11 to 27 post-immunization days) also produced beneficial effects by reducing the development of clinical signs and the expression of pro-inflammatory proteins [95]. In a clinical study conducted on 29 patients with Relapsing-Remitting Multiple Sclerosis (RR-MS), oral supplementation with PEA-um (600 mg/day for 12 months, added to subcutaneous Interferon (IFN)-β1a), beside relieving pain at IFN-β1a injection site, significantly reduced the plasma concentration of inflammatory cytokines (IFN-γ, IL-17, TNF-α) [96]. The quality of life of supplemented patients was also improved compared to the placebo-treated group, as assessed with MSQoL-54—Multiple Sclerosis Quality of Life 54 questionnaire [96]. 

### 3.4. Amyotrophic Lateral Sclerosis

Amyotrophic lateral sclerosis (ALS) is a deadly neurodegenerative disease characterized by the ongoing degeneration of motor neurons, which leads to progressive paralysis of skeletal muscle and death in 3–5 years after diagnosis. Neuroinflammation is currently considered a highly important driving force [162,163]. In a clinical case of a patient affected by sporadic ALS, oral supplementation with PEA-um improved the respiratory and motor functions, accompanied by the appearance of muscle tone, plausibly because of the control of neuroinflammation [97]. More recently, a broad clinical study performed on 64 patients suffering from ALS has shown that dietary administration of PEA-um (600 mg twice daily for 6 months) added to standard therapy (i.e., riluzole) significantly slowed down the decline in pulmonary function as measured by forced vital capacity (FVC), and lowered the severity of ALS symptoms, compared to patients treated with riluzole alone [98]. It is also noteworthy that a short-term add-on dietary PEA-um (600 mg twice daily for 1 week) proved to reduce the level of disability and improve muscular response to fatigue in 22 patients with myasthenia gravis (MG), as assessed by Repetitive Nerve Stimulation and Quantitative MG score [99].

### 3.5. Autism Spectrum Disorders

Autism spectrum disorders (ASD) are a range of heterogeneous neurodevelopmental conditions defined by repetitive behaviors as well as deficits in socialization and communication. The affected patients frequently present altered levels of cytokines, including an increase of IL-1β, IL-6, IL-8, IFN-γ, eotaxin, and monocyte chemotactic protein-1 (MCP-1) [164], confirming the role of neuroinflammation in the etiology of autism [165]. The first case report on PEA-um in ASD dealt with two children (aged 13 and 15) [102]. Both patients had severe comprehension problems and difficulty expressing themselves, and suffered from behavioral disorders [102]. The add-on administration of PEA-um (600 mg twice daily for 3 months) to the standard therapy led to a clear improvement in the expressive, relational, and cognitive-behavioral abilities, in both patients [102]. Moreover, in a translational study it was found that co-ultraPEA-Lut (1 mg/kg for 2 weeks or 3 months) ameliorated social and nonsocial behaviors in a murine model of valproic acid-induced autistic behaviors, and benefited a male child aged 10 affected by ASD (700 mg + 70 mg bid) [100]. In particular, after one-year supplementation, the child experienced reduced ASD-associated behavioral problems, especially in the area of social skills and anxiety [100]. In the BTBR mouse model of idiopathic autism, dietary supplementation with PEA-um (30 mg/kg for 10 days) was recently shown to revert the altered behavioral phenotype through the activation of PPAR-α and reduce the inflammatory state in the hippocampus [101]. In addition, improvement of the epithelial barrier integrity and microbiota composition in the gut was also detected, suggesting an involvement of microbiota-gut-brain axis [101].

## 4. Pre-Clinical and Clinical Effects of PEA in Micronized and Co-Micronized Formulations in Pain Syndromes Sustained by Neuroinflammation

### 4.1. Acute and Chronic Pain

Accumulating evidence suggests that neuroinflammation—accompanied by non-neuronal cell activation and pro-inflammatory factor release—is a major contributor to pain states [7,166,167]. Several studies report the protective effects of PEA in different murine models of neuropathic pain (NP). In particular, in mice with chronic constriction injury (CCI) of the sciatic nerve, PEA relieved thermal hyperalgesia as well as mechanical allodynia, and reduced sensitizing factors (such as nerve growth factor, NGF) [103,104,105]. Moreover, the administration of this lipid amide prevented the reduction of myelin sheet thickness and axonal diameter, and reduced edema and macrophage infiltration [103,104,105]. The anti-nociceptive functions exerted by PEA depended, at least in part, on the down-regulation of non-neuronal cells, as shown by the delayed mast cell recruitment and decreased mast cell degranulation in the sciatic nerve, as well as reduced microglia activation at the spinal level [104]. Interestingly, the control of microglia activation was also shown to mediate the effect of PEA in formalin-induced neuropathic pain [106]. Moreover, PEA improved pain-related behaviors in the spared nerve injury (SNI) model of NP [107] and oxaliplatin-induced neuropathy [108]. One of the mechanisms sustaining the anti-nociceptive function of PEA, at least in models of acute and persistent inflammatory pain (i.e., formalin test and carrageenan-induced paw edema) is thought to be a PPAR-α receptor-mediated increase of neurosteroidogenesis [109]. Micronized and ultramicronized formulations of PEA, PEA-m, and PEA-um respectively, and their effect on acute and neuropathic pain were also investigated. In this regard, interesting results came from a study in which PEA-m was administered to rats (30 mg/kg for 11 days) concurrently treated with morphine [110]. The results demonstrated that PEA-m attenuated the development of tolerance to morphine, doubling the number of days morphine was effective, and preventing morphine-induced hyperplasia of microglia and astrocytes, thus suggesting the usefulness of adding PEA-m to opioid-based therapies [110]. The efficacy of PEA-um formulation was also demonstrated in the SNI model [111]. In particular, PEA-um (10 mg/kg once a day, started 15 days after the sham or SNI surgery and lasted for 15 days) was able to relieve thermal hyperalgesia and mechanical allodynia [111]. In addition, it improved cognitive impairments and neurogenesis, and restored the level of glutamate as well as the expression of phosphorylated metabotropic glutamate receptor 1 (GluR1) subunits [111]. In a mice model of tibia fracture derived pain, which resembles the clinical features of acute complex regional pain syndrome type 1 (CRPS-I), a new formulation of PEA-m together with PEA-um (300 mg/kg + 600 mg/kg for 28 days) improved the fracture regeneration and reduced the thermal nociception and mechanical hyperalgesia [112]. The effect was found to partly depend on the control of mast cell density and the decrease of NGF and cytokines expression [112]. Moreover, a recent study investigated the synergistic effect of PEA-um with paracetamol on neuroinflammation and neuropathic pain in a rat model of sciatic nerve injury [113]. Combining PEA-um to paracetamol at ineffective or, at most, marginally effective doses (5 mg/kg and 30 mg/kg, respectively) for a 14-day regimen after sciatic nerve injury induction relieved hyperalgesia, protected nerve fibers against histological damage and cell apoptosis, down-modulated endoneural mast cells, and reduced NGF expression and serum cytokine levels [113]. Recently, PEA-m has been shown to relieve one of the most common form of acute pain, i.e., post-operative pain [114]. In particular, oral administration of PEA-m (10 mg/kg) at different time points, either before and after a surgical paw incision in rats proved to significantly reduce hyperalgesia [114]. Control of surgery-induced mast cell hyperplasia at the paw level and NGF increase at the spinal level were also shown, together with the return to nearly normal levels of microglia and astrocyte markers respectively [114]. On the clinical side, several observational studies have been conducted on patients with chronic pain of different etiologies. In particular, a pilot study has been performed on 610 patients with chronic pain associated to different pathological conditions, whose pain was not adequately controlled by standard analgesic therapies [115]. Dietary administration of PEA-um (600 mg twice daily for 3 weeks followed by single daily administration for 4 weeks, in addition to standard analgesic drugs or as a single intervention) showed a significant decrease of pain severity assessed by the Numeric Rating Scale (NRS) [115]. All patients administered PEA-um completed the study without reporting adverse effects [115]. The effect of PEA-um-based FSMP has also been evaluated in 30 patients with diabetic or traumatic chronic neuropathic pain [116]. Patients given PEA-um (1200 mg/die for 40 days) showed a significant improvement of pain and paraesthesia/dysesthesia scores, assessed by Visual Analogue Scale (VAS) and Neuropathic Pain Symptom Inventory (NPSI), respectively. In addition, PEA-um-administered patients experienced a better quality of life, as evaluated by means of the Five-Dimensional Health Questionnaire (EQ-5D) [116]. A further study has recently been performed on 155 patients with lower back pain related to nonsurgical lumbar radiculopathy. They were given acetaminophen/codeine (500 mg + 30 mg/die) for 7 days, and then changed to PEA-um (1200 mg/die) for 30 days. The treatment regimen relieved pain in all patients with mild pain (VAS from 3–4 to 1) and 75% of those with moderate pain (VAS from 5–6 to 2) [117]. Patients who did not experience pain or disability improvement had a second cycle with PEA-um (600 mg/die for 30 days) followed by acetaminophen/codeine for 30 days, which was found to relieve symptoms in all patients with moderate pain [117]. Given together with tapentadol a FSMP based on PEA-um (600 mg twice daily for 6 months) provided a superior effect on pain relief compared to tapentadol only in 55 patients with low back pain [118]. Moreover, patients in the combined treatment group recorded a lower analgesic dose requirement and lesser disability compared to patients in the tapentadol only group [118]. A further investigation has been carried out on 35 patients affected by failed back surgery syndrome, who were orally administered a FSMP based on PEA-um (1200 mg/die for the first month followed by 600 mg/die for the second month) as an add-on dietary intervention to tapentadol and pregabalin [119]. A significant adjunctive effect was observed in pain relief (pain intensity was measured by VAS) with no serious side effects being recorded in this as well as the previously described studies [117,118,119]. Recently, the benefit of combining a PEA-um based FSMP with rehabilitative therapy has also been studied in patients with chronic low back pain [120]. The study enrolled 120 patients who suffered from lumbosciatica (95) and lumbocruralgia (25) pain caused by multiple herniated discs in the lumbar spine [120]. PEA-um (600 mg twice a day for 40 days) was added to a standard analgesic regimen and combined with a 20-day session of daily functional rehabilitation and decontracting massage [120]. During the study period, patients experienced a significant reduction of pain severity (scored using the Numeric Rating Scale) accompanied by improvements in the physical and mental components of quality of life (evaluated with the SF-36 questionnaire), as well as in disability for low back pain (assessed by Oswestry Disability Questionnaire) [120]. Oral supplementation with PEA-um (600 mg twice daily) has also been evaluated in an open controlled study on 42 patients waiting for carpal tunnel syndrome surgery and suffering from sleep disorders and painful symptoms [121]. During the pre-surgery period a significant improvement of the sleep quality with an increase of continuous time sleep (measured by Pittsburgh Sleep Quality Index), and mitigation of the painful stimuli (assessed by NRS), were recorded in PEA-um group compared to untreated patients [121]. It is finally worth noting that a post-hoc analysis of a controlled study on over 600 patients with lower back pain (i.e., a mixed pain condition) has recently demonstrated that the higher the probability of suffering from neuropathic pain, the better the treatment outcome following PEA-m oral administration [168]. The analysis yielded a Number Needed to Treat (NNT) value of 1.7 for PEA-m 600 mg daily, which is considerably better than that of first-line drugs, with tricyclic antidepressants yielding a score of 3.5, serotonin-norepinephrine reuptake inhibitors of 6.4, gabapentin of 7.2, and pregabalin of 7.7 [168].

### 4.2. Chronic Pelvic Pain

When not appropriately regulated, neuroinflammation importantly contributes to the pathogenesis of endometriosis, a chronic estrogen-dependent gynecological disorder that often causes dysmenorrhea and pelvic pain [169].

A study in a rat model of endometriosis has recently shown that PEA-um (10 mg/kg for 25 days) reduced the number and duration of pain crises and cyst diameter [122]. Moreover, the number of mast cells and vessels as well as the expression of NGF in cysts and dorsal root ganglia were also significantly reduced [122]. These results suggested a potential clinical utility of PEA-um for the control of chronic pelvic pain associated with endometriosis [122]. Accordingly, in a preliminary observational study 4 patients with endometriosis-related pain were administered a FSMP containing PEA-m and polydatin (400 mg + 40 mg twice daily) for 90 days [123]. All patients showed pain relief (measured by VAS) as early as 1 month after starting the supplement and a reduction in the use of analgesic drugs that were previously employed for pain control [123]. Likewise, 24 women with symptoms of severe pelvic pain and suspected endometriosis experienced significant pain relief (assessed by VAS) after 90-day supplementation with a FSMP containing PEA-m and polydatin (400 mg + 40 mg twice daily) [124]. Quality of life was also improved and the use of non-steroidal anti-inflammatory drugs (NSAIDs) decreased during the study [124]. Similar results were observed after the 3-month administration of the same FSMP (same dose) in 61 subjects with chronic pelvic pain related to endometriosis after laparoscopic conservative surgery [125]. A significant reduction in dysmenorrhea, dyspareunia, and pelvic pain (measured by VAS) was recorded, the effect being nearly equal to that exerted by celecoxib (a NSAIDS drug) [125]. Moreover, the studied FSMP proved to be safe, thus suggesting it could be a valuable alternative in those patients who cannot take analgesic drugs [125]. The same combination of PEA-m and polydatin has also been used in another clinical study performed on 47 women with chronic pelvic pain due to endometriosis, who were divided in two groups based on the endometriosis site: recto-vaginal septum or ovary [126]. In both groups the dietary supplementation showed a significant reduction in chronic pelvic pain, dysmenorrhea, dyspareunia, and dyschezia (evaluated by VAS) already after 30 days [126]. A further study evaluated the effect of a 10-day regimen with FSMP containing PEA-m and polydatin (400 mg + 40 mg once a day, taken from the 24th day of cycle) compared to placebo. Two groups of 110 young patients each (age 16–24 years) with primary dysmenorrhea were included [127]. Pain relief was shown to be superior in the FSMP group compared to the placebo group [127]. Finally, an interesting finding comes from a recently published study performed on 30 women with the diagnosis of endometriosis and pregnancy desire, administered PEA-um twice daily for 10 days followed by PEA-m/polydatin twice daily for 80 days [128]. The severity of chronic pelvic pain, dyspareunia, dysmenorrhea, dyschezia, and dysuria was evaluated using VAS, while the quality of life and women’s psychological well-being were evaluated with 36-Item Short Form Health Survey Questionnaire and Symptom Check list-90 Questionnaire, respectively [128]. At the end of the study, all patients showed a significant improvement in painful symptoms, quality of life and psychological well-being, and did not record any grave side effect, proving that the study FSMP is particularly suitable for women with pregnancy desire and without other infertility factors [128].

### 4.3. Migraine Pain

Mast cells involved in neuroinflammation in the brain are considered key players in migraine pathophysiology [14].

The effect of a PEA-um based FSMP in the dietary management of migraine has been initially evaluated in a clinical case of nummular migraine [129]. The patient, a 57-year-old woman suffering from superficial cranial pain since the last 10 years was administered PEA-um (600 mg/day) after a one-month treatment with topiramate (50 mg × 2/day tablet), had lead only a minor improvement on pain scales (6 on VAS, 6 on NRS, and severe pain on VRS) [129]. Two months later, a clear decrease of pain was recorded (2 on VAS, 3 on NRS, and moderate-to-mild pain on VRS) and a progressing scaling of topiramate up to 25% of the original dose was decided. At the last follow-up (4 months after PEA-um was initiated) pain was importantly relieved (1 on VAS, 1 on NRS, and no or mild pain on VRS), with no adverse effects being recorded [129]. A single blind study was then conducted to evaluate both the safety and the efficacy of PEA-um (1200 mg/day) in 40 patients suffering of migraine with aura treated with NSAIDs (ibuprofen, diclofenac sodium, or nimesuilde) [130].

At the end of the study (3 months later) a significant pain relief (evaluated either as VAS score and number of attacks/month) was found in the group of patients taking PEA-um and NSAIDs, which was superior to that recorded in patients managed with NSAIDs only [130]. Moreover, unlike the control group, patients treated with PEA-um reduced the NSAIDs consumption throughout the study [130]. Finally, an open-label study has recently been conducted on 70 pediatric patients (with mean age of 10.3  ±  2.7, 24.5% male and 75.5% female) with a diagnosis of migraine without aura [131]. The patients received PEA-um (at the dose of 600 mg/day) for three months, and the headache attack frequency (AF) and attack intensity (AI) were measured at baseline and at the end of the study [131]. After three months, 63.9% of patients recorded a reduction of the headache AF by >50%; the number of monthly attacks, headache AI, percentage of patients with severe attacks and monthly assumption of drugs for the attack were significantly reduced [131]. In this study, only 1 patient recorded mild side effect consisting of nausea and vomiting [131]. 

### 4.4. Fibromyalgia 

Fibromyalgia (FM) is a particularly disabling musculoskeletal disease characterized by widespread chronic pain, muscle stiffness and fatigue, often refractory to common analgesic drugs. There is considerable evidence of the involvement of neuroinflammation in FM pathogenesis [170,171,172]. In a observational study conducted on 80 patients with FM, 30-day administration of a FSMP containing PEA-um (600 mg/bid) followed by two-month supplementation with PEA-m (300 mg/bid) in addition to the standard therapy (duloxetine and pregabalin) showed a significant and greater improvement in pain intensity (assessed by VAS) and positive tender points, compared to the control group treated with duloxetine and pregabalin only [132]. None of the patients enrolled in this study recorded adverse side effects [132]. Similar results have been reported in another retrospective observational study recently performed on 407 patients with the diagnosis of FM, who received PEA-um (600 mg/day) regardless of the concurrent pharmacological therapy (add-on intervention) [133]. The results showed that 359 patients recorded an improvement in the pain score (measured by VAS) and quality of life (assessed by Fibromyalgia Impact Questionnaire, FIQ), with only 36 patients reporting adverse events principally of gastrointestinal type (diarrhea, dyspepsia, bloating, constipation, and vomiting) [133].

## 5. Conclusions

In the light of increasing evidence for a key role of uncontrolled neuroinflammation in the pathogenesis of common and disabling disorders, targeting non-neuronal cells is emerging as a promising therapeutic strategy. PEA is an endogenous fatty acid amide with protective functions mainly exerted through the down-regulation of non-neuronal cells (such as mast cells, microglia and astrocytes) at both central and peripheral level. The shift toward a homeodynamic phenotype exerted by the prophylactic administration of PEA confirms its protective role, i.e., the ability to prepare cells to successfully cope with incoming perturbations [173,174,175]. The data reviewed in the present paper highlight the effectiveness and safety of PEA in controlling neuroinflammation, once it has been administered in formulations with adequate bioavailability, i.e., micronized or ultra-micronized forms [57,76]. Moreover, the endogenous nature, the occurrence in common food sources [72,73,74] (Table 1) and the physiological functions described so far sustain the value of supplementing PEA to meet the increased requirements in the course of clinical conditions sustained by neuroinflammation. In the words of the Nobel Prize winner Rita Levi-Montalcini, “*The observed effects of Palmitoylethanolamide appear to reflect the consequences of supplying the tissue with a sufficient quantity of its physiological regulator of cellular homeostasis*” [141]. Particular FSMPs containing PEA-m and PEA-um (alone or in combination with compounds endowed with important antioxidant properties, such as Luteolin and polydatin) have been developed in recent years [29]. As discussed in the present review paper, they have been repeatedly evaluated in clinical trials and collectively resulted safe and effective in patients with neurodegenerative and neurological disorders, and pain syndromes sustained by neuroinflammation, especially if utilized in the context of a multimodal pharmacotherapy. This is particularly relevant for frail patients (i.e., with declined physiological reserve and vulnerability to adverse outcomes) given that PEA-m and PEA-um based FSMPs resulted to synergize conventional therapies and decrease the effective dose of drugs that are usually associated with frequent or even serious adverse effects [87,118,123,124,130]. All that said, PEA-based FSMPs may be deservedly considered part of a new and promising nutritional approach to disorders sustained be neuroinflammation. The recent FDA approval for the adjunct use of PEA-um in patients affected by one the most severe forms of neuroinflammation, i.e., COVID-19, provides a further proof-of-concept in this respect [https://www.biospace.com/article/releases/fsd-pharma-begins-phase-2-clinical-trial-to-evaluate-fsd201-for-the-treatment-of-hospitalized-covid-19-patients/].

## Figures and Tables

**Table 1:** PEA content in plant and animal derived foods. * 110 ± 32.3 lactation days; ^(§)^ on a dry weight basis.

Food	PEA Concentrations (ng/g of Fresh Weight)	Reference
Bovine milk	~0.25	[65]
Elk milk	1.81	[65]
Human breast milk	8.98 ± 3.35 nM	[58]
Human breast milk *	23.4 ± 7.2 nM	[47]
Alfalfa (*Medicago sativa*)	1150	[64]
Garden pea (*Pisum sativum*)	103	[64]
Black-eyed pea (*Vigna unguiculata*)	138	[64]
Common bean (*Phaseolus vulgaris*)	53.5	[64]
Peanut (*Arachis hypogaea*)	3730	[64]
Toasted peanuts	10,900	[72]
Soybean (*Glycine max*)	6720	[64]
Soy lecithin	950,000	[63]
Corn (*Zea mays*)	200	[63]
Tomato (*Lycopersicon esculentum*)	100	[63]
Walnut	253.3 ± 14.6 ^(§)^	[73]
Refined wheat flour	803.7 ± 0.26 ^(§)^	[73]
Coffee powder	897.7 ± 15.1 ^(§)^	[73]
Margarine	302.3 ± 5.35 ^(§)^	[73]
Eggs	~80 ^(§)^	[73]
Parmigiano cheese	~50 ^(§)^	[73]
Mozzarella cheese	~40 ^(§)^	[73]
Tuna fish	~20 ^(§)^	[73]
Codfish	~60 ^(§)^	[73]
Anchovies	~40 ^(§)^	[73]
Chicken	~120 ^(§)^	[73]
Salami	~100 ^(§)^	[73]
Mussel (*Mytilus galloprovincialis*)	21.0 ± 3.0	[74]
Clams (*Venus verrucosa*)	39.2 ± 6.1	[74]
Clams (*Tapes decussatus*)	58.8 ± 6.2	[74]
Clams (*Callista chione*)	28.4 ± 4.5	[74]
Oyster (*Crassostrea* sp)	16.4 ± 1.6	[74]

**Table 2:** Pre-clinical and clinical effects of PEA in micronized and co-micronized formulations on neuroinflammation associated with neurodegenerative and neurological disorders. Abbreviations: Aβ, beta amyloid; ALS, amyotrophic lateral sclerosis; ASD, autism spectrum disorders; bid, twice a day; co-ultraPEA-Lut, PEA co-ultra-micronized with Luteolin; EAE, experimental autoimmune encephalomyelitis; FSMP, Foods for Special Medical Purposes; i.h., intra-hippocampal; i.p., intra-peritoneal; MCI, mild cognitive impairment; MG, myasthenia gravis; mos., months; MPTP, 1-methyl-4-phenyl-1 2 3 6-tetrahydropyridine; PEA, palmitoylethanolamide; PEA-m: micronized PEA; PEA-um: ultra-micronized PEA; PD, Parkinson’s disease; p.o.; oral; RoA, routes of administration; RR-MS, relapsing-remitting multiple sclerosis; s.c., subcutaneous; SL, sublingual; tabs, tablets; tid, three times a day; TMEV-IDD, Theiler’s Murine Encephalomyelitis Virus-Induced Demyelinating Disease; wks, weeks; 3×Tg-AD, triple transgenic Alzheimer’s disease; 6-OHDA: 6-hydroxydopamine.

Disease/Model/Condition			Subject	Formulation	RoA	Main Effect	Ref.
Parkinson’s Disease	Pre-clinical	MPTP	Mice	PEA (10 mg/kg, 7 days)	i.p.	Neuroprotective	[84]
		MPTP	Mice	co-ultraPEA-Lut (1 mg/kg, 7 days)	i.p.	Neuroprotective	[85]
		MPTP	Mice	PEA-m pre-treatment (10 mg/kg, 60 days)	p.o. (gavage)	Neuroprotective	[80]
		6-OHDA	Mice	PEA (3–10-30 mg/kg, 28 days)	s.c.	Neuroprotective	[86]
	Clinical	PD	30 Patients	PEA-um (600 mg/bid, 3 mos. + 600 mg/die, 12 mos.) + Levodopa	p.o.	Effective add-on therapy	[87]
Alzheimer’s Disease	Pre-clinical	Aβ 25–35 peptide	Mice	PEA (3–10-30 mg/kg, 1–2 wks)	s.c.	Neuroprotective	[88]
		Aβ 1–42 peptide	Rats	PEA (10 mg/kg, 7 days)	i.p.	Neuroprotective	[89]
		3 × Tg-AD	Mice	PEA-um (10 mg/kg, 3 mos.)	s.c.	Neuroprotective	[90,91]
		3 × Tg-AD	Mice	PEA-um (100 mg/kg, 3 mos.)	p.o. (mixed to food)	Neuroprotective	[77]
		Aβ 1–42 peptide	Rats	co-ultraPEA-Lut (5 mg/kg, 2 wks)	i.p.	Neuroprotective	[92]
	Clinical	MCI	1 Patient	FSMP based on co-ultraPEA-Lut (700 + 70 mg/die, 9 mos.)	p.o. (SL μ-granules)	Neuro-psychological amelioration	[93]
Multiple Sclerosis	Pre-clinical	TMEV-IDD	Mice	PEA (5 mg/kg for 10 days)	i.p.	Neuroprotective	[94]
		EAE	Mice	co-ultraPEA-Lut (5 mg/kg, 17 days)	i.p.	Neuroprotective	[95]
	Clinical	RR-MS	29 Patients	FSMP based on PEA-um (600 mg/day, 12 mos.) + IFN-β1a	p.o. (tabs)	Pain relief and quality of life amelioration	[96]
Amyotrophic Lateral Sclerosis	Clinical	Sporadic ALS	1 Patient	FSMP based on PEA-um (600 mg/tid SL + 1200 mg/die, 40 days)	p.o. (SL μ-granules + tabs)	Respiratory and motor function amelioration	[97]
		ALS	64 Patients	FSMP based on PEA-um (600 mg/bid, 6 mos.) + riluzole	p.o. (tabs)	Slowdown of decline in pulmonary function and symptom severity	[98]
		MG	22 Patients	FSMP based on PEA-um (600 mg/bid, 1 wk)	p.o.	Disability reduction and amelioration of muscular response to fatigue	[99]
Autism	Pre-clinical	Valproic acid	Mice	co-ultraPEA-Lut (1 mg/kg, 2 wks or 3 mos.)	p.o. (gavage)	Social and non-social behavior improvement	[100]
		BTBR T + tf/J	Mice	PEA-um (10–30 mg/kg, 10 days)	i.p.	Microbiota-gut-brain axis improvement	[101]
	Clinical	ASD	2 Children	FSMP based on PEA-um (600 mg/bid, 3 mos.)	p.o.	Amelioration of expressive, relational and cognitive-behavioral ability	[102]
		ASD	1 Child	FSMP based on co-ultraPEA-Lut (700 + 70 mg/bid, 1 year)	p.o.	Reduction of worsening in social skills and anxiety	[100]

**Table 3:** Pre-clinical and clinical effects of PEA in micronized and co-micronized formulations in pain syndromes sustained by neuroinflammation. Abbreviations: bid, twice a day; CCI, chronic constriction injury; FSMP, food for special medical purpose; i.p., intra-peritoneal; i.pl., intra-plantar; NP, neurophatic pain; p.o., oral. PEA-m, micronized PEA; PEA-um, ultra-micronized PEA; PEA, palmitoylethanolamide; s.c., subcutaneous; SNI, spared nerve injury.

Disease/Model/Condition			Subject	Formulation	RoA	Main Effect	Ref.
Acute and chronic pain	Pre-clinical	CCI	Mice	PEA (30 mg/kg, 14 days)	s.c. or i.p.	Anti-neuroinflammatory and anti-nociceptive	[103,104,105]
		Formalin	Mice	PEA (5–10 mg/kg, 7 days)	i.p.	Anti-neuroinflammatory and anti-nociceptive	[106]
		SNI	Mice	PEA (10 mg/kg, 15–30 days)	i.p.	Anti-nociceptive	[107]
		Oxaliplatin	Rats	PEA (30 mg/kg, 21 days)	i.p.	Anti-nociceptive	[108]
		Formalin and carrageenan-	Mice	PEA (50 μg/10 μL)	i.pl.	Anti-nociceptive	[109]
		Morphine	Rats	PEA-m (30 mg/kg, 11 days) + morphine	s.c.	Attenuation of development in tolerance to morphine	[110]
		SNI	Mice	PEA-um (10 mg/kg, 15 days)	i.p.	Anti-nociceptive and improvement of cognitive-decline	[111]
		Tibia fracture	Mice	PEA-m and PEA-um (300 mg/kg and 600 mg/kg, 28 days)	p.o.	Anti-nociceptive and improvement of fracture regeneration	[112]
		Sciatic nerve injury	Rats	PEA-um (5 mg/kg, 14 days) + paracetamol	p.o. by gavage	Anti-neuroinflammatory and anti-nociceptive	[113]
		Post-operative pain	Rats	PEA-m (10 mg/kg at different time points before/after incision)	p.o. by gavage	Anti-neuroinflammatory and anti-nociceptive	[114]
	Clinical	Chronic pain associated to different pathological conditions	610 patients	FSMP based on PEA-um (600 mg/bid, 3 weeks and 600 mg/die, 4 weeks) + analgesic drugs	p.o.	Reduction of pain severity	[115]
		Diabetic or traumatic chronic NP	30 patients	FSMP based on PEA-um (1200 mg/die, 40 days)	p.o. (sachet or tablet)	Reduction of pain and paraesthesia/dysesthesia scores. Quality of life amelioration	[116]
		Low back pain related to nonsurgical lumbar radiculopathy	155 patients	First cycle: Acetaminophen/codeine for 7 days + FSMP based on PEA-um (1200 mg/die, 30 days). Second cycle: FSMP based on PEA-um (600 mg/die, 30 days) + acetaminophen/codeine for 30 days	p.o.	First cycle: pain relief in all patients with mild pain and in 75% with moderate pain. Second cycle: Pain relief and improvement of disability in all patients with moderate pain. Improvement of disability in 74% of patients with severe pain	[117]
		Chronic low back pain	55 patients	FSMP based on PEA-um (600 mg/bid, 6 months) + tapentadol	p.o.	Pain relief. Reduction of disability and analgesic dose requirement.	[118]
		Failed back surgery syndrome	35 patients	FSMP based on PEA-um (1200 mg/die for the first month + 600 mg/die for the second month) + tapentadol and pregabalin	p.o.	Pain relief	[119]
		Lumbosciatica (95) and lumbocruralgia (25) pain	120 patients	FSMP based on PEA-um (600 mg/bid, 20 days followed by 600 mg of PEA-um/die, 40 days) + analgesic drugs + rehabilitation and decontracting massage	p.o.	Reduction of pain severity and disability for low back pain. Quality of life amelioration	[120]
		Waiting for carpal tunnel syndrome surgery and affected by sleep disorders and painful symptoms	42 patients	FSMP based on PEA-um (600 mg/bid during the pre- and post-surgery periods)	p.o.	Amelioration of sleep quality and mitigation of painful stimuli	[121]
Chronic pelvic pain	Pre-clinical	Endometriosis plus ureteral calculosis	Rats	PEA-um (10 mg/kg, 25 days)	p.o.	Anti-neuroinflammatory and reduction of the number and duration in pain crises and cyst diameter	[122]
	Clinical	Endometriosis-related pain	4 patients	FSMP based on PEA-m and polydatin (400 mg + 40 mg/bid, 90 days)	p.o.	Pain relief and reduction in the use of analgesic drugs	[123]
		Symptoms of severe pelvic pain and suspected endometriosis	24 women	FSMP based on PEA-m and polydatin (400 mg + 40 mg/bid, 90 days)	p.o.	Pain relief and quality of life amelioration. Reduction in the use of NSAIDs	[124]
		Chronic pelvic pain related to endometriosis after laparoscopic conservative surgery	61 patients	FSMP based on PEA-m and polydatin (400 mg + 40 mg/bid, 90 days)	p.o.	Reduction of dysmenorrhea, dyspareunia and pelvic pain.	[125]
		Chronic pelvic pain due to endometriosis	47 women	FSMP based on PEA-m and polydatin (400 mg + 40 mg/bid, 90 days)	p.o.	Reduction of dysmenorrhea, dyspareunia and pelvic pain.	[126]
		Primary dysmenorrhea	110 young patients	FSMP based on PEA-m and polydatin (400 mg + 40 mg/die taken from the 24th day of cycle for 10 days)	p.o.	Pain relief	[127]
		Diagnosis of endometriosis and pregnancy desire	30 women	FSMP based on PEA-um (600 mg/bid, 10 days) and PEA-m and polydatin (400 mg + 40 mg/bid, 80 days)	p.o.	Amelioration in painful symptoms, quality of life and psychological well-being	[128]
Migraine pain	Clinical	Superficial cranial pain	1 patient	FSMP based on PEA-um (600 mg/day, 4 months) + topiramate	p.o.	Pain relief	[129]
		Migraine with aura	20 patients	FSMP based on PEA-um (1200 mg/die, 3 months) + NSAIDs	p.o.	Pain relief and reduction in the use of NSAIDs	[130]
		Diagnosis of migraine without aura	70 pediatric patients	FSMP based on PEA-um (600 mg/day, 3 months)	p.o.	Reduction of the headache attack frequency by >50% per month in 63.9% of patients.	[131]
Fibromyalgia	Clinical	Fibromyalgia	80 patients	FSMP based on PEA-um (600 mg/bid, 30 days) and PEA-m (300 mg/bid, 2 months) + duloxetine and pregabalin	p.o.	Amelioration in pain intensity	[132]
		Diagnosis of fibromyalgia	407 patients	FSMP based on PEA-um (600 mg/day, add-on treatment)	p.o.	359 patients recorded an amelioration in the pain score and quality of life	[133]
